# National audit of perinatal HIV infections in the UK, 2006–2013: what lessons can be learnt?

**DOI:** 10.1111/hiv.12577

**Published:** 2018-01-16

**Authors:** H Peters, C Thorne, PA Tookey, L Byrne

**Affiliations:** ^1^ Population Policy & Practice Programme UCL Great Ormond Street Institute of Child Health London UK

**Keywords:** HIV, perinatal infection, pregnancy, prevention of mother‐to‐child transmission, UK

## Abstract

**Objectives:**

The aim of the study was to investigate circumstances surrounding perinatal transmissions of HIV (PHIVs) in the UK.

**Methods:**

The National Study of HIV in Pregnancy and Childhood conducts comprehensive surveillance of all pregnancies in women diagnosed with HIV infection and their infants in the UK; reports of all HIV‐diagnosed children are also sought, regardless of country of birth. Children with PHIV born in 2006–2013 and reported by 2014 were included in an audit, with additional data collection via telephone interviews with clinicians involved in each case. Contributing factors for each transmission were identified, and cases described according to main likely contributing factor, by maternal diagnosis timing.

**Results:**

A total of 108 PHIVs were identified. Of the 41 (38%) infants whose mothers were diagnosed before delivery, it is probable that most were infected *in utero*, around 20% intrapartum and 20% through breastfeeding. Timing of transmission was unknown for most children of undiagnosed mothers. For infants born to diagnosed women, the most common contributing factors for transmission were difficulties with engagement and/or antiretroviral therapy (ART) adherence in pregnancy (14 of 41) and late antenatal booking (nine of 41); for the 67 children with undiagnosed mothers, these were decline of HIV testing (28 of 67) and seroconversion (23 of 67). Adverse social circumstances around the time of pregnancy were reported for 53% of women, including uncertain immigration status, housing problems and intimate partner violence. Eight children died, all born to undiagnosed mothers.

**Conclusions:**

Priority areas requiring improvement include reducing incident infections, improving ART adherence and facilitating better engagement in care, with attention to addressing the health inequalities and adverse social situations faced by these women.

## Introduction

There are around 35 000 women living with HIV in the UK and every year around 1200 become pregnant. Pregnant women access antenatal and HIV services through the publicly funded National Health Service, which are free at the point of care for residents. National standards and guidelines for antenatal screening, obstetric care and management of HIV infection in pregnancy are provided by different organizations 1, 2, 3, 4, 5, and there is > 95% uptake of routinely offered antenatal HIV screening 3. Vertical transmission in women diagnosed with HIV infection in the UK declined from 2.1% in 2000–2001 to 0.7% in 2006–2007, 0.5% in 2010–2011 6 and 0.3% in 2012–2014 7, reflecting a universal offer of antenatal screening, high uptake of earlier and effective antiretroviral therapy (ART) and optimized clinical care during pregnancy, at birth and in the postnatal period 6. As these vertical transmission rates demonstrate, there are now very few infections in infants born to women known to be living with HIV. However, there are additional infants with perinatally acquired HIV (PHIV) born in the UK where maternal infection status was not known by the time of delivery. This is of particular concern, in view of the increased risk of serious morbidity and mortality if ART is not started early 8.

The design of the UK and Ireland National Study of HIV in Pregnancy and Childhood (NSHPC) incorporates data collection on both groups of infants with PHIV. Our previous audit of PHIV in England in 2002–2005 highlighted failures in communication, failure to act on suboptimal virological response to ART, and adverse social circumstances which acted as barriers to prevention of mother‐to‐child transmission (MTCT) 9. The question remains: how can we further reduce the number of new paediatric HIV infections in the UK? In a new audit, we have examined the individual circumstances of all children with PHIV born in the UK between 2006 and 2013 to identify missed opportunities for preventing vertical transmission. This project was funded by the UK National Screening Committee specifically to provide recommendations for the antenatal HIV screening programme, as well as to identify trends and common factors between the cases to inform clinical management more widely.

## Methods

The NSHPC seeks comprehensive reporting of all pregnancies in women diagnosed with HIV infection prior to or during their current pregnancy in the UK or Ireland and all infants with *in utero* HIV exposure; in addition, reports of all children diagnosed with HIV infection (< 16 years old) are sought, regardless of country of birth. The core mechanisms are two confidential active reporting schemes: (1) for HIV infection in pregnant women, through a quarterly reporting system with named respondents in every maternity unit; (2) for HIV‐exposed and ‐infected children, through monthly reporting via the British Paediatric Surveillance Unit or directly to the NSHPC from some large clinics with a high case load 10, 11. The NSHPC does not have access to patient names; identifiers used were those provided by reporting clinicians (mainly unique study numbers and dates of birth).

### Study population

All children with PHIV, born in the UK between 1 January 2006 and 31 December 2013 and reported by 31 March 2014, were included. The term ‘case’ is used to describe one mother–child pair.

### Data collection

Two main data sources were utilized: variables collected within routine NSHPC surveillance and already included in the study database (described above) and additional data collected via interviews with clinicians involved in the care of each case. Interviews were conducted by two of the authors (LB and HP). The paediatrician who had reported the case was initially contacted and interviewed. Obstetric respondents were interviewed in all cases where the mother had been diagnosed with HIV infection by delivery, but otherwise the obstetric unit was only contacted if they were aware of the case, or once the paediatric team had informed them [as recommended by the Children's HIV Association (CHIVA)] 9. Interviews with an HIV clinician with knowledge of the case were conducted if required. If the mother/child had been seen at multiple units, all relevant units were contacted. Clinicians informed their answers with accessible hospital records.

Interviews sought information about factors that might have facilitated PHIV transmission. Interviewees were also asked about the presence of uncertain immigration status, housing problems, diagnosed mental health problems, drug/alcohol use, and intimate partner violence, and any other adverse social circumstances or complicating issues. Following the interviews, each case was summarized.

An expert review panel was convened to assess anonymized case summaries, including likely timing of transmission, missed opportunities and common patterns, and to make recommendations to strengthen national PMTCT policy. The panel included clinicians with expertise in managing HIV infection in pregnant women and children (paediatrics, obstetrics, midwifery and HIV medicine/infectious diseases), the Infectious Disease Screening Programme (IDPS) programme director, IDPS clinical advisor, members of the NSHPC and community representatives (see Acknowledgements for membership list).

The expert review panel found that in most cases only one contributing factor was evident; where multiple factors were identified, one was assigned as the likely *main* contributing factor (the factor thought to have been most important, taking into account all available evidence on the timing of maternal and infant infections). The contributing factors identified are defined in Table 1.

**Table 1:** Definitions of contributing factors

Contributing factor	Definition
Woman declined antenatal HIV testing	Clinicians reported that the woman was offered and declined HIV testing in pregnancy
Seroconversion	The woman acquired HIV infection during pregnancy or after delivery after initially testing negative
Problems with engagement or adherence	Clinicians looking after the woman in pregnancy reported that the woman had difficulties attending antenatal appointments (including appointments with their HIV clinicians), and/or adhering to antiretroviral therapy
Postnatal transmission probably attributable to breastfeeding	Clinicians reported evidence that the timing of the transmission was postnatal, in women diagnosed by delivery.
Woman presented late for antenatal care	The woman did not access antenatal care for the first time in the pregnancy until after 24 weeks of gestation
Woman transferred antenatal care provider	The woman was seen at more than one unit for antenatal care
Pre‐term delivery impact on duration of treatment	The infant was delivered pre‐term, and this impacted on the duration of antiretroviral therapy and therefore the woman's viral load at the time of delivery
Problem with antenatal HIV test	The woman was offered and accepted an HIV test in pregnancy, but the result was not available because of a processing or reporting error

### Definitions

Women were classified as ‘diagnosed’ if they had been diagnosed with HIV infection at any time up to delivery and ‘undiagnosed’ if diagnosed after delivery. Baseline viral load (VL) was at diagnosis, or first result reported in pregnancy.

Late booking for antenatal care was defined as first antenatal visit at ≥ 24 weeks of gestation, reflecting British HIV Association (BHIVA) guidelines 1.

Infants with a positive polymerase chain reaction (PCR) within 3 days of birth were classified as having likely *in utero* transmission; infants with a negative PCR within 3 days, then a positive PCR within 6 weeks were classified as having likely intrapartum transmission; infants with a negative PCR after 6 weeks and a positive PCR thereafter were classified as having likely postnatal transmission, according to generally accepted definitions 12.

### Statistical analyses

Data were managed in access 2010 and excel 2013 (Microsoft Corp., Redmond, WA, usa), and analysed using stata version 12.1 (Stata Corp. LP, College Station, TX, USA). Categorical variables were compared using *χ* ^2^ tests or Fisher's exact tests, and medians using Kruskal–Wallis tests. Mortality rates were calculated using the time ‘at risk’ from the child's birthdate to the end of the data collection period or date of death.

## Results

Approximately 9200 live births to HIV‐diagnosed women in the UK in 2006–2013 were reported by April 2014, and 41 of their children were reported to have PHIV. A further 67 children born in the UK during this period, whose mothers had not been diagnosed with HIV infection by delivery, were diagnosed and reported with PHIV.

**Figure 1:**
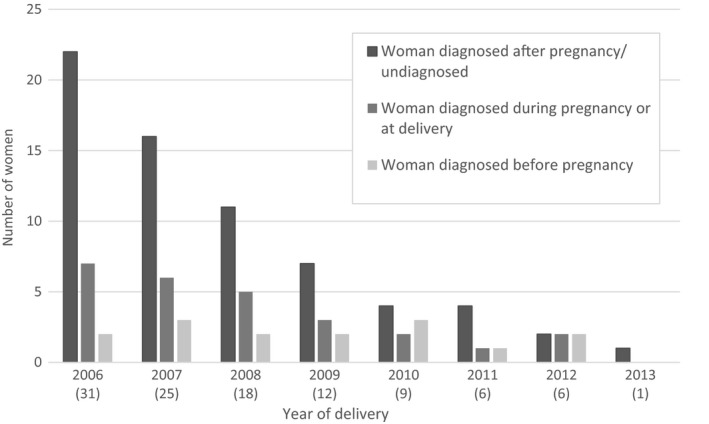
Number of perinatally infected infants, by timing of maternal diagnosis and year of birth (*n *=* *108). Numbers in parentheses are totals.

Median maternal age at delivery was 29 years [interquartile range (IQR) 26–34 years]. Overall, nearly 90% (94 of 108) of the mothers were born abroad (mainly in Africa), with 39% (22 of 57) having lived in the UK for > 5 years (Table 2). The number of infants with PHIV born each year declined during the period under review, particularly those born to undiagnosed mothers (Fig. 1).

**Table 2:** Maternal sociodemographic and HIV diagnosis characteristics (*n *=* *108)

	Women diagnosed by delivery (*n *=* *41)		Women undiagnosed by delivery (*n *=* *67)	
Characteristic	*n*	%	*n*	%
Age (years) at delivery	(*n *=* *41)		(*n *=* *62)	
< 20	3	7	3	5
20–29	20	49	27	44
30–39	17	42	30	48
≥ 40	1	2	2	3
Marital status	(*n *=* *41)		(*n *=* *63)	
Married/cohabiting	30	73	49	78
Separated	5	12	7	11
Single	6	15	7	11
Employment status	(*n *=* *36)		(*n *=* *66)	
Employed (health care)	1	4	11	16
Employed (other)	7	19	22	33
Student	8	22	4	6
Unemployed	20	55	29	43
Partner employment status	(*n *=* *24)		(*n *=* *31)	
Employed	15	63	24	77
Student	5	21	0	0
Unemployed	4	17	7	23
Region of birth	(*n *=* *41)		(*n *=* *67)	
Africa	34	83	51	76
UK	4	10	10	15
Elsewhere in Europe	1	2	4	6
Asia	2	5	1	2
Caribbean	0	0	1	2
Years in UK prior to delivery if born abroad	(*n *=* *27)		(*n *=* *30)	
< 1	6	22	5	17
1–5	10	37	14	47
6–10	7	26	9	30
> 10	4	15	2	7
How or where HIV Identified	(*n *=* *38)		(*n *=* *61)	
Antenatal screening current/previous pregnancy	29	77	–	–
subsequent pregnancy	–	–	6	10
Child found positive	–	–	25	41
Partner found positive	0	0	3	5
Genitourinary medicine clinic	4	10	8	13
Other hospital department	1	3	11	18
Other	4	10	8	13
Mode of HIV acquisition	(*n *=* *38)		(*n *=* *55)	
Heterosexual	36	94	54	98
Injecting drug use	1	3	1	2
Vertical transmission	1	3	0	0

Of the 108 mother–child pairs, 62% had one likely MTCT contributing factor identified, another 22% had two, 5% had three and one had four. In 5%, no contributing factor could be identified and in another 5% of cases minimal information was available. Table 3 shows the total number of women reported with each contributing factor and the number for whom it was the *main* factor.

**Table 3:** Contributing factors in cases of perinatal transmission (*n *=* *108)

	Diagnosed women (*n *=* *41)			Undiagnosed women (*n *=* *67)		
	All contributing factors	*Main* contributing factor		All contributing factors	*Main* contributing factor	
Contributing factor	*n*	*n*	%^a^	*n*	*n*	%^a^
Woman declined antenatal HIV testing	2	–	–	31	28	42
Seroconversion in pregnancy/postnatal period	2	2	5	23	23	34
Engagement/treatment adherence issues	19	14	34	–	–	–
Postnatal transmission probably attributable to breastfeeding	7	7	17	1	1	2
Woman presented late for antenatal care	11	9	22	7	3	4
Woman transferred antenatal care provider	4	–	–	–	–	–
Pre‐term delivery impact on duration of treatment	3	3	7	2	–	–
Problem with antenatal HIV test	1	1	3	8	7	10
No specific contributing factor identified	5	5	12	–	–	–
Missing information	2	0	0	19	5	8
Total		41	100		67	100

a Adjusted to total 100%.

Reporting clinicians were aware that 53% of women had experienced at least one of the specific adverse social circumstances or other complicating issues in pregnancy, including 19% with at least two and 7% with at least three issues. A breakdown of the complicating issues is provided in Table 4.

**Table 4:** Adverse social circumstances and complicating issues for the mother reported at the time of pregnancy (*n *=* *108)

Additional issue	Diagnosed (*n *=* *41)		Undiagnosed (*n *=* *67)	
	*n* a	%^a^	*n* a	%^a^
Uncertain immigration status	12	29	16	24
Housing problems	12	29	18	27
Intimate partner violence	4	10	8	12
Drug/excess alcohol use	4	10	7	10
Diagnosed mental health issue	8	20	6	9
Social service involvement	8	20	9	13
Incarceration/partner incarceration	3	7	3	4
Maternal age < 18 years at delivery	0	–	2	3
Not fluent in English	11	27	5	7

a Some women were reported to have several issues, so *n* totals > 108 (> 100%).

## Infants born to diagnosed women

Among these 41 infants, 26 were born to mothers diagnosed during the current pregnancy (see Table 5 for maternal CD4 counts, VLs and treatment). Just over half were probably infected *in utero* (23 of 41), nearly 20% intrapartum (seven of 41) and 20% in the postnatal period through breast feeding (seven of 41) (timing unclear for four of 41).

**Table 5:** Maternal clinical markers for women diagnosed before or at delivery (*n *=* *41)

	Women diagnosed before pregnancy (*n* = 15)		Women diagnosed during pregnancy or at delivery (*n* = 26)	
	*n*	%	*n*	%
Viral load nearest conception^b^	(*n *=* *14)^a^		(*n *=* *26)	
< 1000	3	21	2	8
1000–99 999	10	71	16	61
≥ 100 000	1	7	8	31
Viral load nearest delivery^b^	(*n *=* *14)^a^		(*n *=* *25)	
< 50	7	50	3	12
50–999	3	21	9	36
1000–99 999	3	21	10	40
≥ 100 000	1	8	3	12
CD4 count nearest conception^c^	(*n *=* *13)^a^		(*n *=* *26)	
< 250	4	31	10	39
250–349	5	38	6	23
350–499	1	8	6	23
≥ 500	3	23	4	15
On ART at conception	(*n *=* *14)^a^			
Yes	6	43	–	–
No	8	57	–	–

a One case of missing information on test results and ART at conception as respondent could not access case notes.

ART, antiretroviral therapy.

b copies/mL

c cells/*μ*L

### Contributing factors

The most common factor contributing to perinatal infection among these 41 cases was problems with engagement and/or ART adherence in pregnancy (recognized in 19 cases). This was the *main* contributing factor in 14 cases (in the remaining five the *main* factor was postnatal transmission, probably due to undisclosed breast feeding in four, and late booking in one). Half were diagnosed before pregnancy. One woman had acquired HIV from her mother. Most (11 of 14) had at least one complicating issue reported, including diagnosed mental health problems (four cases), insecure housing (four) and involvement of social services (three). Reported problems faced by these women included non‐acceptance of diagnosis, concerns over disclosure, treatment side effects and issues swallowing tablets. Interventions ranging from additional support with adherence (from a multidisciplinary approach) to directly observed therapy were provided in all but two cases, where the women (both diagnosed during pregnancy) disengaged from care after diagnosis. All 14 women were prescribed protease inhibitor‐based combination antiretroviral therapy (cART); eight had a VL > 10 000 HIV‐1 RNA copies/mL at delivery. Ten of the 14 infants were delivered by caesarean section (CS); 11 of 14 were probably infected *in utero*, and one intrapartum (timing unknown for two).

Late presentation for antenatal care (documented in 11 cases) was identified as the *main* contributing factor in nine cases. Of these nine, all women were diagnosed antenatally, booking at a median of 36 weeks of gestation (IQR 27, 36 weeks). Five were known to have been travelling or living abroad until late pregnancy (> 30 weeks); four were known to have language communication barriers. Complicating issues among these women included uncertain immigration status (four women), intimate partner violence (two) and insecure housing (two). Eight of these nine cases were classified as *in utero* transmissions (timing unclear in one).

Postnatal transmission, probably as a result of undisclosed breastfeeding, was the *main* contributing factor in seven of 41 cases. In three of seven cases, ART was known to have stopped after delivery, consistent with guidelines at the time. In the remaining four cases, there were adherence issues in pregnancy, with either loss to follow‐up or continuing adherence issues postnatally; in all four cases there were known concerns about disclosure of HIV status to family or friends. All seven women had additional issues reported, including insecure housing (four women), uncertain immigration status (three), social services involvement (one) and mental health issues (one).

Pre‐term delivery was the *main* contributing factor in three of 41 cases: in these, cART was started at a median of 24 weeks, and two delivered at < 32 weeks and one at 32–36 weeks. All three pre‐term deliveries were emergency CS, with VL at delivery of 50–399 copies/mL in two and 1000–10 000 copies/mL in the third. One case was a likely *in utero* transmission, and two were likely intrapartum transmissions.

Two further women were diagnosed with primary HIV infection late in pregnancy (29 and 39 weeks, respectively) following a negative test at booking, and transmissions were likely to have been *in utero* in these cases. In one other case, the *main* contributing factor was a delayed test result (and subsequent initiation of treatment); this transmission probably occurred *in utero*.

In the five remaining cases, despite information being available from clinicians, it was not possible to establish a clear contributing factor. In four of the five cases, transmission probably occurred intrapartum: all had undetectable VL and were on cART before delivery (one spontaneous vaginal delivery and three elective CSs).

## Infants born to undiagnosed women

Of the 67 children born to undiagnosed women, 42 were diagnosed with HIV infection as a consequence of their mother or other family member being diagnosed. The remaining 25 children presented with symptoms at a median of 6 months (IQR 3, 16 months), although in a third of cases (eight of 25) an HIV test was not offered at first presentation despite the child having an HIV indicator condition.

Timing of HIV acquisition could not be estimated for nearly 90% of these 67 children. There was one likely intrapartum and two likely *in utero* transmissions; these infants were tested soon after birth following postnatal maternal diagnosis.

### Contributing factors

In nearly half (31 of 67) of all perinatal infections where the mother was undiagnosed in pregnancy, antenatal HIV screening had been declined, with this identified as the *main* contributing factor in 28 of 31 cases. These 28 women all delivered in 2006–2010, most in 2006–2007. The reason for the decline was available in 12 cases, and included needle phobia, not feeling at risk of HIV infection, confidentiality concerns, wanting to discuss with partner, and feeling unable to cope with a positive result. In six of the 28 cases there was documentation of a re‐offer; three of the six involved an experienced clinician. In two cases it transpired that the woman had not disclosed a previous positive HIV test. In the majority of these cases (20 of 28), at least one complicating issue was reported.

Of the 23 of 67 women who acquired HIV infection after testing negative early in pregnancy, 16 delivered in 2006–2009 and seven in 2010–2013. Median gestation at antenatal test was 12 weeks (IQR 9–17 weeks); the median interval to the mother's first positive HIV test was 6.2 months after delivery (IQR 2.6–22 months). In this group, one current male partner tested negative and 17 partners were known to be HIV positive or subsequently tested positive. Of the four men known to have HIV infection at the time of the pregnancy, two had not disclosed their status.

In eight of the 67 undiagnosed women, there was a problem in processing the HIV test; all these deliveries occurred before 2009. For a further three women, the *main* contributing factor for transmission was late antenatal booking (after 30 weeks of gestation), with two not arriving in the UK until late pregnancy; two were diagnosed in the delivery suite. All three were reported to have uncertain immigration status, along with other complicating issues.

### Missing information

In nine cases, the relevant antenatal unit was unaware of the maternal diagnosis, so could not be contacted (in three cases the child or mother had died). In one case, the antenatal unit could not identify the mother, and archived antenatal notes were inaccessible in another. Antenatal notes were unavailable to the interviewee for 18 of 53 women where the main contributing factor was declined antenatal testing or seroconversion.

### Child mortality

Overall, eight children (all born to undiagnosed women) were known to have died by the end of the study period: one from complications following pre‐term delivery and seven of HIV‐related causes; six died under the age of 6 months, and two at 18–24 months. The proportion of children who died by age 2 years was 8% overall, and 12% in those born to undiagnosed women. The crude mortality rate was 1.4 deaths per 100 child‐years overall (95% confidence interval 0.67–2.70); 2.2 per 100 child‐years for children born to undiagnosed women (95% confidence interval 1.1–4.4).

## Discussion

We identified 108 children with PHIV born in the UK between 2006 and 2013 by April 2014, of whom around 60% were born to mothers undiagnosed at delivery; this is slightly lower than reported in our earlier audit 9. Of the 41 children of diagnosed mothers, the majority were infected *in utero*. Our audit demonstrated that at least half of women were experiencing adverse social circumstances, and in nearly a third of cases multiple factors were reported. This is a minimum estimate as information was limited in about a fifth of cases. At least one key factor likely to have contributed directly to HIV transmission was identified in the vast majority of cases. The most common were decline of HIV testing in pregnancy (accounting for nearly half of undiagnosed women) and seroconversion (around a quarter). Other factors compromising prevention of mother‐to‐child transmission (PMTCT) included engagement/adherence to ART, late antenatal booking, breastfeeding and pre‐term delivery, as has been found among perinatal transmissions in diagnosed women occurring elsewhere across Europe 13.

The number of children with PHIV born each year declined from 31 children in 2006 to six in 2012 and one in 2013. This is consistent with the declining MTCT rate 7, although national rates are based on PHIV infants born to diagnosed mothers. Ascertainment of HIV status in pregnancy is believed to exceed 95%, but the number of women delivering with undiagnosed HIV infection is unknown. Prompt diagnosis of infants born to diagnosed women is a priority, in order to start ART immediately in the rare situation of PHIV, reflected by the median age at diagnosis of one month reported here. Diagnosis of children born to undiagnosed women was mainly precipitated by an HIV diagnosis in a family member, and occurred at a median 7.5 months. Therefore, there might be some children with as yet undiagnosed PHIV born in this audit period. Nevertheless, the number of infants born to undiagnosed women and diagnosed before their first birthday also fell, suggesting a reduction in the overall number of pregnant and breastfeeding women living with undiagnosed HIV infection, consistent with national trends in adults living with HIV 14.

In untreated non‐breastfeeding women, most transmissions occur intrapartum 12, but here, as previously reported 6, over half of infected infants with diagnosed mothers acquired HIV infection *in utero*. The mechanisms of *in utero* transmission are incompletely understood, but high maternal VL and delayed ART initiation increase risk 15, 16.

A fifth of infants born to diagnosed women probably acquired HIV infection postnatally through undisclosed breastfeeding. Although women with HIV infection in the UK are still recommended to avoid breastfeeding, clinicians are now advised to support women who choose to breastfeed with close monitoring to minimize the risk of transmission 1. None of the mothers of infants with postnatal acquisition disclosed a desire or intention to breastfeed; however, all delivered in an era predating the current guidelines when there was the strong recommendation to avoid breastfeeding, and clinicians could refer to child protection services if they suspected breastfeeding. Studies in African settings with mothers on cART (with unknown VLs) have shown transmission rates of 0.3–3% 17, 18, 19, 20, 21, 22, 23, and UK breastfeeding guidance for mothers with HIV infection has evolved in line with this evidence. Avoidance of breastfeeding can come at great personal cost 24 and financial support for replacement feeding for women with HIV infection in the UK is patchy 25. Our findings highlight the risks of unsupported breastfeeding; clinical teams must facilitate an open discussion, elicit barriers to replacement feeding, and support women to minimize the risk of transmission.

The 2010 and 2016, national antenatal screening standards recommended re‐offering testing to women declining an HIV test at booking 2, 26. We found that at least 40% of undiagnosed women who declined a test were not re‐offered testing; however, these predated full national implementation of the 2010 standards and it is encouraging that no declines were observed after 2010.

Seroconversions in pregnancy or postnatally after an earlier negative antenatal HIV test occurred in around a quarter of women, including some with a partner with known HIV infection, similar to the previous audit 9. MTCT rates are higher in women acquiring HIV infection antenatally or postnatally as a result of high levels of viral replication 27. Repeat third trimester screening for women testing negative at booking is unlikely to significantly reduce transmissions as it would not identify maternal infections acquired late in pregnancy or postnatally 28, 29. However, repeat testing should be offered to women known to be at continuing risk of acquiring HIV 1 and pre‐exposure prophylaxis (PrEP) could be offered in such cases. HIV testing for male partners in antenatal settings is challenging, with low uptake and structural barriers reported 30, 31.

People living with HIV face many potential barriers to optimal ART adherence, such as fear of disclosure, complex regimens, and work and family responsibilities 32. A meta‐analysis found that only 72% of pregnant women with HIV infection had adequate adherence, and this fell postnatally 33. Protective factors include strong social support, acceptance of diagnosis and a daily routine 32. Guidelines recommend management by an experienced multidisciplinary team with general practitioner and health visitor involvement 1, with access to peer support. Women may require additional services such as counselling, social services and advocacy.

Among diagnosed women, the *main* factor contributing to transmission was late antenatal booking, this being the main factor in nearly a quarter of these women. Pregnant women with HIV infection in the UK are accessing antenatal and HIV care earlier than in the past, but 36% booked after 13 weeks of gestation in 2012–2014, with women from sub‐Saharan Africa and parous women at increased risk 24, 34. Multiple barriers to timely antenatal booking have been identified among black and minority ethnic women and addressing these will require a range of interventions and approaches 35.

In five cases it was not possible to identify any factor contributing to the transmission. Although for women who achieve an undetectable VL by delivery the risk of MTCT is very low 6, clinicians must be aware that these cases still occur.

A strength of this study was the near ‘complete’ picture of PHIV in UK‐born infants 36 as a result of our national surveillance design. However, there were limitations too: the additional data collection was retrospective, subject to recall bias and limited to the perspectives of the clinicians. Difficulties in accessing case notes in some instances may have led to underestimation of complicating issues. Enhanced surveillance of PHIV is now conducted contemporaneously within routine NSHPC data collection to improve data quality and minimize missing data.

## Conclusions

The MTCT rate in the UK in diagnosed women is at an all‐time low and reports of PHIV in infants born to undiagnosed women have also declined. This audit provides important insights into contemporary cases of PHIV transmission in the UK that could inform future policy and practice, with areas of clinical practice requiring improvement identified. Priorities include promoting earlier booking in key obstetric populations, reducing incident infections in pregnancy and during breastfeeding and improving adherence and service engagement in pregnancy and postpartum, including addressing the health inequalities and adverse social situations that these women face. Our findings highlight the importance of multidisciplinary care and peer support for all women. Further research, including qualitative studies, is needed for example to explore how partner testing within antenatal services and/or PrEP use in pregnancy could be improved, to investigate barriers to care and to monitor outcomes in women with HIV infection who choose to breastfeed.
